# Crystal structure of *N*-(3-hy­droxy­phenyl)succinimide

**DOI:** 10.1107/S1600536814016328

**Published:** 2014-08-01

**Authors:** P. A. Suchetan, S. Naveen, N. K. Lokanath, S. Sreenivasa

**Affiliations:** a Department of Studies and Research in Chemistry, U.C.S., Tumkur University, Tumkur, Karnataka 572 103, India; b Institution of Excellence, Vijnana Bhavan, University of Mysore, Manasagangotri, Mysore 570023, India; c Department of Studies in Physics, University of Mysore, Manasagangotri, Mysore, India; d Department of Studies and Research in Chemistry, Tumkur University, Tumkur, Karnataka 572 103, India

**Keywords:** crystal structure, succinimide, hydrogen bonding

## Abstract

In the title compound, C_10_H_9_NO_3_, the dihedral angle between the benzene and pyrrolidine rings is 53.9 (1)°. In the crystal, mol­ecules are linked through strong O—H⋯O hydrogen bonds into zigzag *C*(8) chains running along [010]. The chains are linked by C—H⋯π inter­actions forming sheets lying parallel to (100).

## Related literature   

For the crystal structures of the 3-methyl and 3-chloro derivatives of *N*-phenyl­succinimide, see: Saraswathi *et al.* (2010 ▶, 2011 ▶).
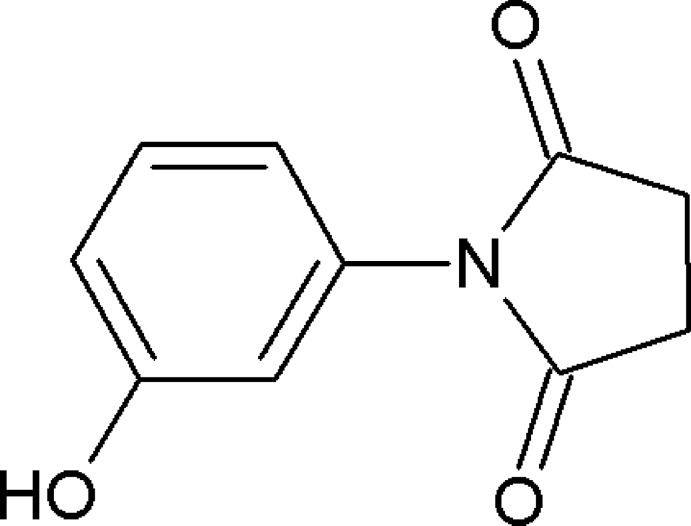


## Experimental   

### Crystal data  

C_10_H_9_NO_3_
*M* *_r_* = 191.18
Monoclinic, 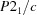

*a* = 11.432 (2) Å
*b* = 7.6567 (14) Å
*c* = 10.115 (2) Å
β = 98.688 (7)°
*V* = 875.2 (3) Å^3^
*Z* = 4
Cu *K*α radiation
μ = 0.91 mm^−1^
*T* = 293 K
0.42 × 0.31 × 0.19 mm

### Data collection  

Bruker APEXII diffractometer
Absorption correction: multi-scan (*SADABS*; Bruker, 2009 ▶) *T*_min_ = 0.749, *T*_max_ = 0.841
3786 measured reflections
1234 independent reflections
1207 reflections with *I* > 2σ(*I*)
*R*_int_ = 0.028
θ_max_ = 60.0°

### Refinement  

*R*[*F* ^2^ > 2σ(*F* ^2^)] = 0.044
*wR*(*F* ^2^) = 0.140
*S* = 1.20
1234 reflections
131 parameters
H atoms treated by a mixture of independent and constrained refinement
Δρ_max_ = 0.16 e Å^−3^
Δρ_min_ = −0.23 e Å^−3^

### 

Data collection: *APEX2* (Bruker, 2009 ▶); cell refinement: *APEX2* and *SAINT-Plus* (Bruker, 2009 ▶); data reduction: *SAINT-Plus* and *XPREP* (Bruker, 2009 ▶); program(s) used to solve structure: *SHELXS97* (Sheldrick, 2008 ▶); program(s) used to refine structure: *SHELXL97* (Sheldrick, 2008 ▶); molecular graphics: *Mercury* (Macrae *et al.*, 2008 ▶); software used to prepare material for publication: *SHELXL97*.

## Supplementary Material

Crystal structure: contains datablock(s) I. DOI: 10.1107/S1600536814016328/su2752sup1.cif

Structure factors: contains datablock(s) I. DOI: 10.1107/S1600536814016328/su2752Isup2.hkl

Supporting information file. DOI: 10.1107/S1600536814016328/su2752Isup3.cml

**Fig. 1.**
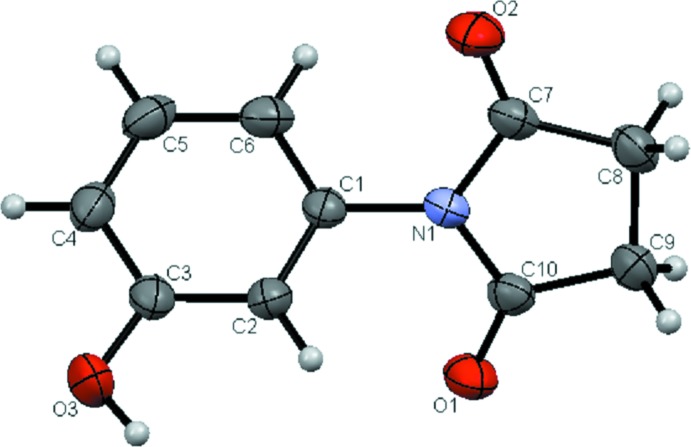
Molecular structure of the title molecule, with atom labeling. Displacement ellipsoids are drawn at the 50% probability level.

. DOI: 10.1107/S1600536814016328/su2752fig1.tif
Mol­ecular structure of the title mol­ecule, with atom labeling. Displacement ellipsoids are drawn at the 50% probability level.

**Fig. 2.**
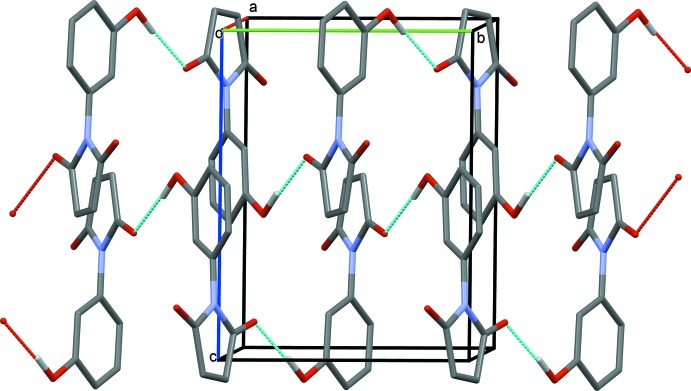
A view along the a axis of the crystal packing of the title compound, showing the formation of the zigzag C(8) chains through O—H···O hydrogen bonds (dashed lines; see Table 1 for details).

. DOI: 10.1107/S1600536814016328/su2752fig2.tif
A view along the a axis of the crystal packing of the title compound, showing the formation of the zigzag C(8) chains through O—H⋯O hydrogen bonds (dashed lines; see Table 1 for details).

**Fig. 3.**
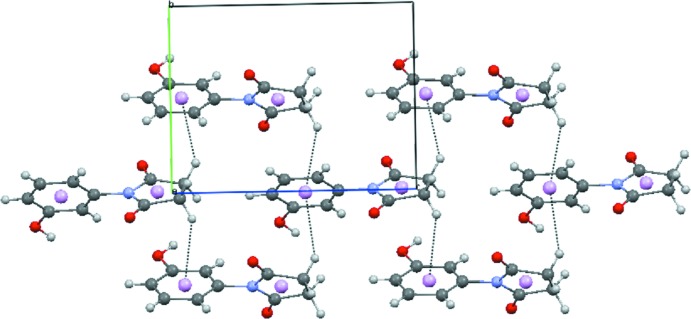
A partial view along the a axis of the crystal packing of the title compound, showing the C—H···π interactions (dashed lines; see Table 1 for details).

. DOI: 10.1107/S1600536814016328/su2752fig3.tif
A partial view along the a axis of the crystal packing of the title compound, showing the C—H⋯π inter­actions (dashed lines; see Table 1 for details).

CCDC reference: 1013858

Additional supporting information:  crystallographic information; 3D view; checkCIF report

## Figures and Tables

**Table 1:** Hydrogen-bond geometry (Å, °) *Cg* is the centroid of the C1–C6 benzene ring.

*D*—H⋯*A*	*D*—H	H⋯*A*	*D*⋯*A*	*D*—H⋯*A*
O3—H3⋯O1^i^	0.94 (4)	1.82 (4)	2.758 (2)	175 (4)
C8—H8*A*⋯*Cg* ^ii^	0.97	2.83	3.613 (3)	138
C9—H9*A*⋯*Cg* ^ii^	0.97	2.79	3.579 (3)	139

Symmetry codes: (i) 
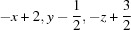
; (ii) 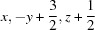.

## supplementary crystallographic information

### S1. Introduction

As a part of a study on the effect of ring and side-chain substitutions on *N*-(Aryl)-succinimides (Saraswathi *et al.*, 2010,2011), the title compound has been synthesized and we report herein on its crystal structure.

### S2.1. Synthesis and crystallization

The title compound was prepared according to the reported procedure (Saraswathi *et al.*, 2010,2011). Colourless prisms of the title compound were obtained by slow evaporation of an aqueous methano­lic solution at room temperature.

### S2.2. Refinement

Crystal data, data collection and structure refinement details are summarized in Table 2. The hydroxyl H atom was located in a difference Fourier map and freely refined. The C-bound H atoms were positioned with idealized geometry and treated as riding atoms: C—H = 0.93-0.97 Å with U_iso_(H) = 1.2U_eq_(C).

### S3. Results and discussion

The title molecule, Fig. 1, is non-planar with the benzene and pyrrolidine [r.m.s. deviation = 0.039 Å] rings tilted at 53.9 (1) °. This is close to the values of 52.5 (1) and 59.5 (1) ° observed in *N*-(3-methyl­phenyl)-succinimide (Saraswathi *et al.* 2010) and *N*-(3-chloro­phenyl)-succinimide (Saraswathi *et al.* 2011).

In the crystal, molecules are linked through strong O—H···O hydrogen bonds into zigzag C(8) chains running along [010] (Table 1 and Fig. 2). The chains are linked by C—H···π inter­actions (Table 1 Fig. 3) forming sheets lying parallel to (100).

### Crystal data

**Table:**

C_10_H_9_NO_3_	Prism
*M**_r_* = 191.18	*D*_x_ = 1.451 Mg m^−^^3^
Monoclinic, *P*2_1_/*c*	Melting point: 393 K
Hall symbol: -P 2ybc	Cu *K*α radiation, λ = 1.54178 Å
*a* = 11.432 (2) Å	Cell parameters from 25 reflections
*b* = 7.6567 (14) Å	θ = 3.9–60.0°
*c* = 10.115 (2) Å	µ = 0.91 mm^−^^1^
β = 98.688 (7)°	*T* = 293 K
*V* = 875.2 (3) Å^3^	Prism, colourless
*Z* = 4	0.42 × 0.31 × 0.19 mm
*F*(000) = 400	

### Data collection

**Table:**

Bruker APEXII diffractometer	1234 independent reflections
Radiation source: fine-focus sealed tube	1207 reflections with *I* > 2σ(*I*)
Graphite monochromator	*R*_int_ = 0.028
phi and φ scans	θ_max_ = 60.0°, θ_min_ = 3.9°
Absorption correction: multi-scan (*SADABS*; Bruker, 2009)	*h* = −12→12
*T*_min_ = 0.749, *T*_max_ = 0.841	*k* = −7→8
3786 measured reflections	*l* = −5→11

### Refinement

**Table:**

Refinement on *F*^2^	Primary atom site location: structure-invariant direct methods
Least-squares matrix: full	Secondary atom site location: difference Fourier map
*R*[*F*^2^ > 2σ(*F*^2^)] = 0.044	Hydrogen site location: inferred from neighbouring sites
*wR*(*F*^2^) = 0.140	H atoms treated by a mixture of independent and constrained refinement
*S* = 1.20	*w* = 1/[σ^2^(*F*_o_^2^) + (0.0766*P*)^2^ + 0.2946*P*] where *P* = (*F*_o_^2^ + 2*F*_c_^2^)/3
1234 reflections	(Δ/σ)_max_ < 0.001
131 parameters	Δρ_max_ = 0.16 e Å^−^^3^
0 restraints	Δρ_min_ = −0.23 e Å^−^^3^

### Special details

**Table:**

Geometry. Bond distances, angles etc. have been calculated using the rounded fractional coordinates. All su's are estimated from the variances of the (full) variance-covariance matrix. The cell esds are taken into account in the estimation of distances, angles and torsion angles
Refinement. Refinement of *F*^2^ against ALL reflections. The weighted *R*-factor *wR* and goodness of fit *S* are based on *F*^2^, conventional *R*-factors *R* are based on *F*, with *F* set to zero for negative *F*^2^. The threshold expression of *F*^2^ > 2σ(*F*^2^) is used only for calculating *R*-factors(gt) *etc*. and is not relevant to the choice of reflections for refinement. *R*-factors based on *F*^2^ are statistically about twice as large as those based on *F*, and *R*- factors based on ALL data will be even larger.

### Fractional atomic coordinates and isotropic or equivalent isotropic displacement parameters (Å^2^)

**Table:**

	*x*	*y*	*z*	*U*_iso_*/*U*_eq_	
O1	0.91904 (12)	0.1492 (2)	0.88087 (15)	0.0436 (5)	
O2	0.56202 (12)	−0.1251 (2)	0.82562 (16)	0.0482 (5)	
O3	0.92280 (13)	−0.1661 (2)	0.43867 (15)	0.0454 (5)	
N1	0.74293 (13)	0.0058 (2)	0.82293 (16)	0.0293 (5)	
C1	0.74308 (15)	−0.0001 (2)	0.68070 (19)	0.0281 (6)	
C2	0.83725 (16)	−0.0782 (2)	0.63281 (18)	0.0300 (6)	
C3	0.83605 (17)	−0.0861 (3)	0.49542 (19)	0.0326 (6)	
C4	0.74209 (18)	−0.0147 (3)	0.4099 (2)	0.0391 (7)	
C5	0.64917 (18)	0.0627 (3)	0.4603 (2)	0.0407 (7)	
C6	0.64839 (17)	0.0704 (3)	0.5970 (2)	0.0361 (6)	
C7	0.65000 (16)	−0.0592 (2)	0.8850 (2)	0.0327 (6)	
C8	0.68144 (17)	−0.0292 (3)	1.0320 (2)	0.0374 (7)	
C9	0.80476 (18)	0.0493 (3)	1.0518 (2)	0.0367 (6)	
C10	0.83298 (16)	0.0764 (2)	0.91334 (19)	0.0313 (6)	
H2	0.90020	−0.12450	0.69140	0.0360*	
H3	0.978 (3)	−0.223 (5)	0.502 (4)	0.088 (10)*	
H4	0.74170	−0.01900	0.31800	0.0470*	
H5	0.58650	0.11000	0.40200	0.0490*	
H6	0.58570	0.12170	0.63140	0.0430*	
H8A	0.68090	−0.13840	1.08050	0.0450*	
H8B	0.62560	0.05040	1.06340	0.0450*	
H9A	0.80610	0.15940	1.09940	0.0440*	
H9B	0.86130	−0.02960	1.10190	0.0440*	

### Atomic displacement parameters (Å^2^)

**Table:**

	*U*^11^	*U*^22^	*U*^33^	*U*^12^	*U*^13^	*U*^23^
O1	0.0306 (8)	0.0573 (10)	0.0443 (9)	−0.0123 (7)	0.0103 (7)	−0.0083 (7)
O2	0.0306 (8)	0.0641 (10)	0.0501 (9)	−0.0141 (7)	0.0066 (7)	0.0068 (8)
O3	0.0417 (9)	0.0634 (10)	0.0328 (9)	0.0051 (7)	0.0110 (7)	−0.0054 (7)
N1	0.0224 (8)	0.0360 (9)	0.0305 (9)	−0.0012 (6)	0.0071 (6)	0.0000 (6)
C1	0.0248 (10)	0.0305 (10)	0.0290 (10)	−0.0052 (7)	0.0045 (8)	0.0009 (7)
C2	0.0248 (10)	0.0362 (11)	0.0283 (10)	−0.0014 (8)	0.0021 (8)	0.0010 (8)
C3	0.0302 (10)	0.0375 (11)	0.0304 (10)	−0.0074 (8)	0.0060 (8)	−0.0009 (8)
C4	0.0377 (12)	0.0478 (13)	0.0299 (10)	−0.0099 (9)	−0.0010 (9)	0.0044 (9)
C5	0.0308 (11)	0.0470 (13)	0.0406 (12)	−0.0039 (9)	−0.0067 (9)	0.0116 (9)
C6	0.0241 (10)	0.0386 (11)	0.0452 (12)	−0.0010 (8)	0.0043 (8)	0.0051 (9)
C7	0.0250 (10)	0.0340 (10)	0.0405 (11)	0.0024 (8)	0.0100 (9)	0.0054 (8)
C8	0.0349 (11)	0.0431 (12)	0.0371 (11)	0.0054 (8)	0.0151 (9)	0.0020 (8)
C9	0.0333 (11)	0.0447 (11)	0.0333 (11)	0.0060 (9)	0.0085 (8)	−0.0043 (9)
C10	0.0250 (10)	0.0345 (11)	0.0353 (11)	0.0028 (8)	0.0072 (8)	−0.0048 (8)

### Geometric parameters (Å, º)

**Table:**

O1—C10	1.218 (2)	C5—C6	1.385 (3)
O2—C7	1.202 (2)	C7—C8	1.494 (3)
O3—C3	1.364 (3)	C8—C9	1.518 (3)
O3—H3	0.94 (4)	C9—C10	1.498 (3)
N1—C1	1.440 (2)	C2—H2	0.9300
N1—C7	1.404 (2)	C4—H4	0.9300
N1—C10	1.380 (2)	C5—H5	0.9300
C1—C2	1.382 (2)	C6—H6	0.9300
C1—C6	1.380 (3)	C8—H8A	0.9700
C2—C3	1.389 (3)	C8—H8B	0.9700
C3—C4	1.386 (3)	C9—H9A	0.9700
C4—C5	1.380 (3)	C9—H9B	0.9700
			
C3—O3—H3	112 (2)	N1—C10—C9	108.71 (16)
C1—N1—C10	124.14 (15)	O1—C10—N1	123.55 (18)
C7—N1—C10	112.43 (16)	C1—C2—H2	121.00
C1—N1—C7	123.43 (15)	C3—C2—H2	121.00
N1—C1—C6	118.80 (16)	C3—C4—H4	120.00
C2—C1—C6	122.34 (18)	C5—C4—H4	120.00
N1—C1—C2	118.85 (16)	C4—C5—H5	120.00
C1—C2—C3	118.63 (17)	C6—C5—H5	120.00
O3—C3—C4	117.31 (17)	C1—C6—H6	121.00
C2—C3—C4	119.80 (18)	C5—C6—H6	121.00
O3—C3—C2	122.87 (18)	C7—C8—H8A	111.00
C3—C4—C5	120.42 (19)	C7—C8—H8B	111.00
C4—C5—C6	120.57 (19)	C9—C8—H8A	111.00
C1—C6—C5	118.24 (19)	C9—C8—H8B	111.00
O2—C7—N1	123.91 (18)	H8A—C8—H8B	109.00
N1—C7—C8	107.82 (16)	C8—C9—H9A	111.00
O2—C7—C8	128.27 (18)	C8—C9—H9B	111.00
C7—C8—C9	105.74 (16)	C10—C9—H9A	111.00
C8—C9—C10	104.97 (16)	C10—C9—H9B	111.00
O1—C10—C9	127.74 (18)	H9A—C9—H9B	109.00
			
C7—N1—C1—C2	−125.04 (18)	N1—C1—C2—C3	178.96 (16)
C10—N1—C1—C2	55.4 (2)	C2—C1—C6—C5	−0.4 (3)
C7—N1—C1—C6	54.1 (2)	C1—C2—C3—C4	0.7 (3)
C10—N1—C1—C6	−125.46 (19)	C1—C2—C3—O3	−177.55 (17)
C7—N1—C10—C9	3.7 (2)	C2—C3—C4—C5	−0.6 (3)
C1—N1—C10—O1	4.0 (3)	O3—C3—C4—C5	177.7 (2)
C7—N1—C10—O1	−175.62 (16)	C3—C4—C5—C6	0.1 (3)
C1—N1—C7—O2	−0.3 (3)	C4—C5—C6—C1	0.4 (3)
C10—N1—C7—C8	−0.1 (2)	O2—C7—C8—C9	177.14 (19)
C10—N1—C7—O2	179.37 (17)	N1—C7—C8—C9	−3.4 (2)
C1—N1—C10—C9	−176.69 (16)	C7—C8—C9—C10	5.3 (2)
C1—N1—C7—C8	−179.75 (16)	C8—C9—C10—O1	173.70 (18)
C6—C1—C2—C3	−0.2 (3)	C8—C9—C10—N1	−5.6 (2)
N1—C1—C6—C5	−179.51 (18)		

### Hydrogen-bond geometry (Å, º)

Cg is the centroid of the C1–C6 benzene ring.

**Table:**

*D*—H···*A*	*D*—H	H···*A*	*D*···*A*	*D*—H···*A*
O3—H3···O1^i^	0.94 (4)	1.82 (4)	2.758 (2)	175 (4)
C8—H8*A*···*Cg*^ii^	0.97	2.83	3.613 (3)	138
C9—H9*A*···*Cg*^ii^	0.97	2.79	3.579 (3)	139

Symmetry codes: (i) −*x*+2, *y*−1/2, −*z*+3/2; (ii) *x*, −*y*+3/2, *z*+1/2.
