# Supplementary figures and images for: Crystal structure of N-(3-hy­droxy­phenyl)succinimide
Source: Acta Crystallogr Sect E Struct Rep Online. 2014 Aug 1;70(Pt 9):o927. doi: 10.1107/S1600536814016328 (PMC4186091; doi:10.1107/S1600536814016328)

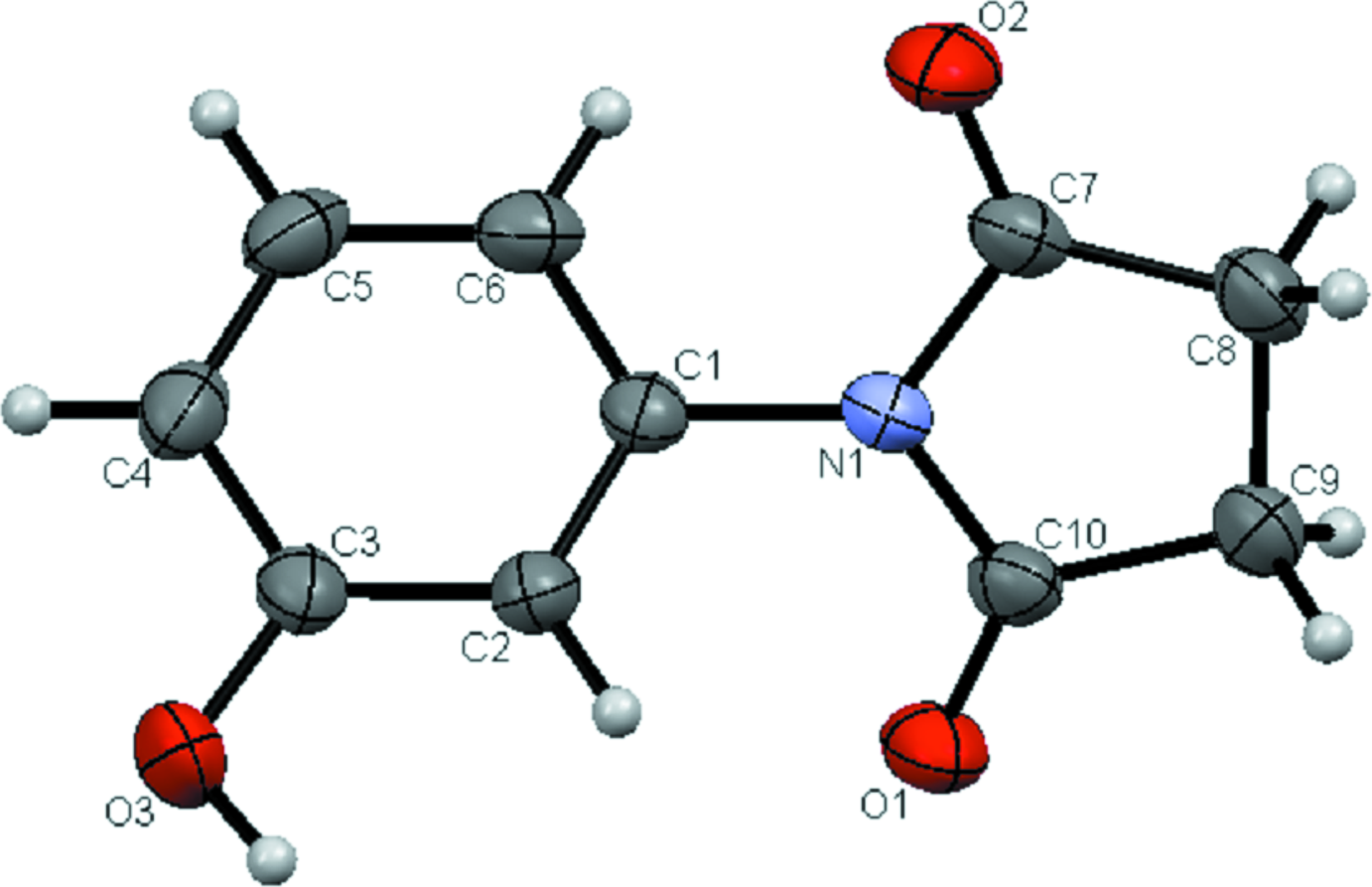

Supplement: Supplementary file 4 [file e-70-0o927-fig1.tif]

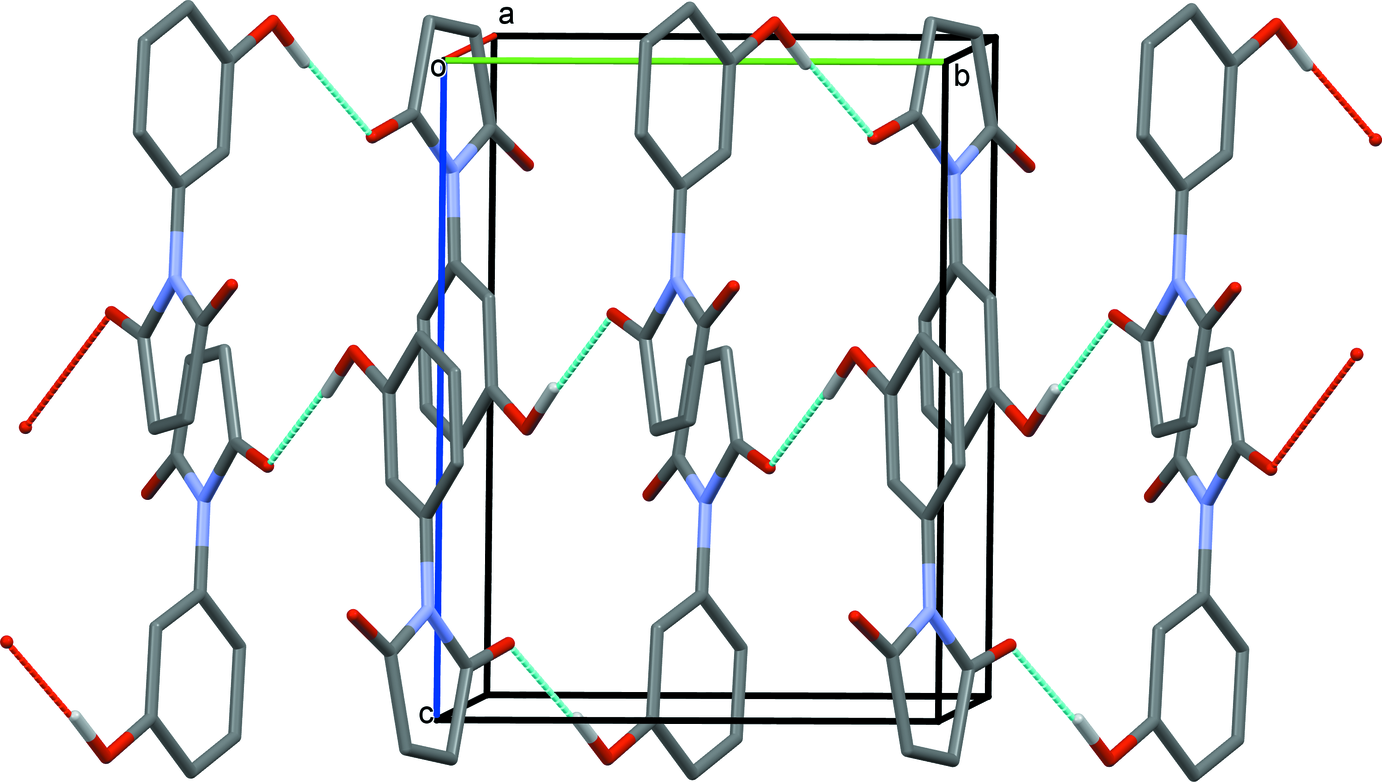

Supplement: Supplementary file 5 [file e-70-0o927-fig2.tif]

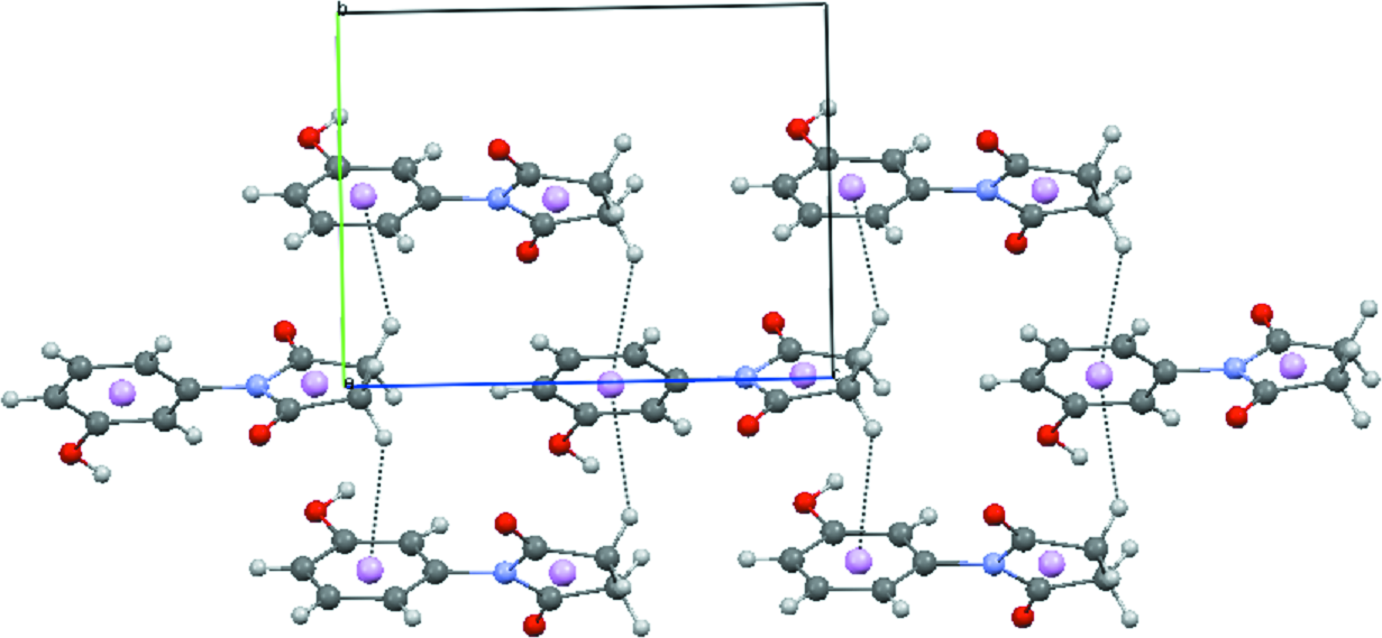

Supplement: Supplementary file 6 [file e-70-0o927-fig3.tif]
